# Testimony on a successful lab protocol to disrupt *Chlorella vulgaris* microalga cell wall

**DOI:** 10.1371/journal.pone.0268565

**Published:** 2022-05-19

**Authors:** Paula A. Lopes, Diogo Coelho, José A. M. Prates

**Affiliations:** 1 CIISA - Centro de Investigação Interdisciplinar em Sanidade Animal, Faculdade de Medicina Veterinária, Universidade de Lisboa, Lisboa, Portugal; 2 Laboratório Associado para Ciência Animal e Veterinária (AL4AnimalS), Lisboa, Portugal; Karl-Franzens-Universitat Graz, AUSTRIA

## Abstract

Over the last decades, microalgae have gained popularity due to demand for novel environmental green solutions and development of innovative mass-production sources for multiple processes, including animal feed and human diet, turning microalgae into an exquisite candidate for several ecofriendly technologies. Notwithstanding, there is a catch. Most species of microalgae, as the case of common *Chlorella vulgaris* (*C*. *vulgaris*) display a recalcitrant cell wall, characterized by a complex matrix of polysaccharides and glycoproteins, which constitutes a major barrier for monogastric species digestibility and extraction of inner valuable nutritional compounds. To overcome this limitation, the development of feed enzymes, in particular Carbohydrate-Active enZymes (CAZymes) with capacity to disrupt *C*. *vulgaris* cell wall may contribute to improve the bioavailability of these microalgae compounds in monogastric diets, namely at high levels of incorporation. In order to disclosure novel combination of feed enzymes to disrupt *C*. *vulgaris* cell wall, a lab protocol was implemented by our research team containing the following key steps: after microalgae cultivation and having available a repertoire of two hundred pre-selected CAZymes produced by high-throughput technology, the step 1 is the individual screening of the most functional enzymes on disrupting *C*. *vulgaris* cell wall (versus a control, defined as the microalgae suspension incubated with PBS) and the determination of reducing sugars released by the 3,5-dinitrosalicylic acid (DNSA) method; step 2 concerns on finding the best CAZymes cocktail, testing the synergistic effect of enzymes, to disrupt *C*. *vulgaris* cell wall (in parallel with running the control) along with characterization of each enzyme thermostability and resistance to proteolytic attack, to which feed enzymes are subjected in the animal gastrointestinal tract; step 3 is the assessment of *C*. *vulgaris* cell wall degradation degree by measuring the amount of reducing sugars released by the DNSA method, fatty acid analysis by gas chromatography (GC) with flame ionization detector (FID), oligosaccharides quantification by high performance liquid chromatography (HPLC) equipped with an electrochemical detector (ECD), protein content by the Kjeldahl method, and various pigments (chlorophylls a and b, and total carotenoids) in the supernatant. In the correspondent residue, we also assessed cellular counting using a Neubauer chamber by direct observation on a bright-field microscope and fluorescence intensity, after staining with Calcofluor White for both control and CAZymes cocktail treatments, on a fluorescence microscope. Beyond animal feed industry with impact on human nutrition, our lab protocol may increase the yield in obtaining valued constituents from *C*. *vulgaris* microalga for other biotechnological industries.

## 1. Microalgae as a promising ecofriendly solution

Microalgae stand now as a proper candidate for various ecofriendly technologies due to their importance, both biologically and economically. The interest in microalgae has increased during the last decades as a result of the need for additional food supplies, among others, contributing to bring back to balance the interplay among environmental, human food and animal feed sustainability [1]. Microalgae popularity is essentially based on the interesting nutritional profiles, production sustainability and inexpensive growth conditions [2]. Microalgae species biodiversity offer a valuable source of nutritional compounds with high added value, such as lipids, proteins, pigments and other antioxidants, vitamins and minerals (reviewed by Madeira et al. [3]), with multiple benefits for human and animal health, due to their antioxidant, antibacterial, antiviral and anti-inflammatory activity. These later aspects have been extensively covered in the literature.

## 2. The biggest drawback on the use of microalgae

The biggest drawback on the use of microalgae is their recalcitrant cell wall. It provides resistance against invaders and/or harsh environmental conditions (such as desiccation during growth), is refractory to breakage and drying, and thus to products removal [4], being largely indigestible by monogastrics. The structural diversity and rigidity of microalga cell walls derive from an extremely diversified and complicated matrix of cross-linked insoluble carbohydrates and glycoproteins that traps valued nutrients, therefore restraining their direct use [5]. On this matter, enzymatic cell wall disruption has shown positive results. It is considered a less energy intensive and more environmentally sustainable approach than the prior available conventional mechanical or chemical methods [6–10]. Exogenous Carbohydrate-Active enZymes (CAZymes), mainly xylanases and β-glucanases, are presently a cost-effective strategy to enhance the nutritional value of cereal-based diets for monogastric livestock species, and directly impact on animal performance and health [11]. Apart from the cost, which seems still prohibitive for industrial applications, CAZymes, individual or combined into a cocktail represent currently the best potential candidates for disruptive treatments. Here is our testimony.

### 2.1. On the design of a successful laboratory strategy to disclosure novel feed enzymes to break up *C*. *vulgaris* cell wall—Methods

The protocol described in this peer-reviewed article is published on protocols.io, https://dx.doi.org/10.17504/protocols.io.dm6gpb9z5lzp/v1 and is included for printing as S1 File with this article.

**Fig 1:**
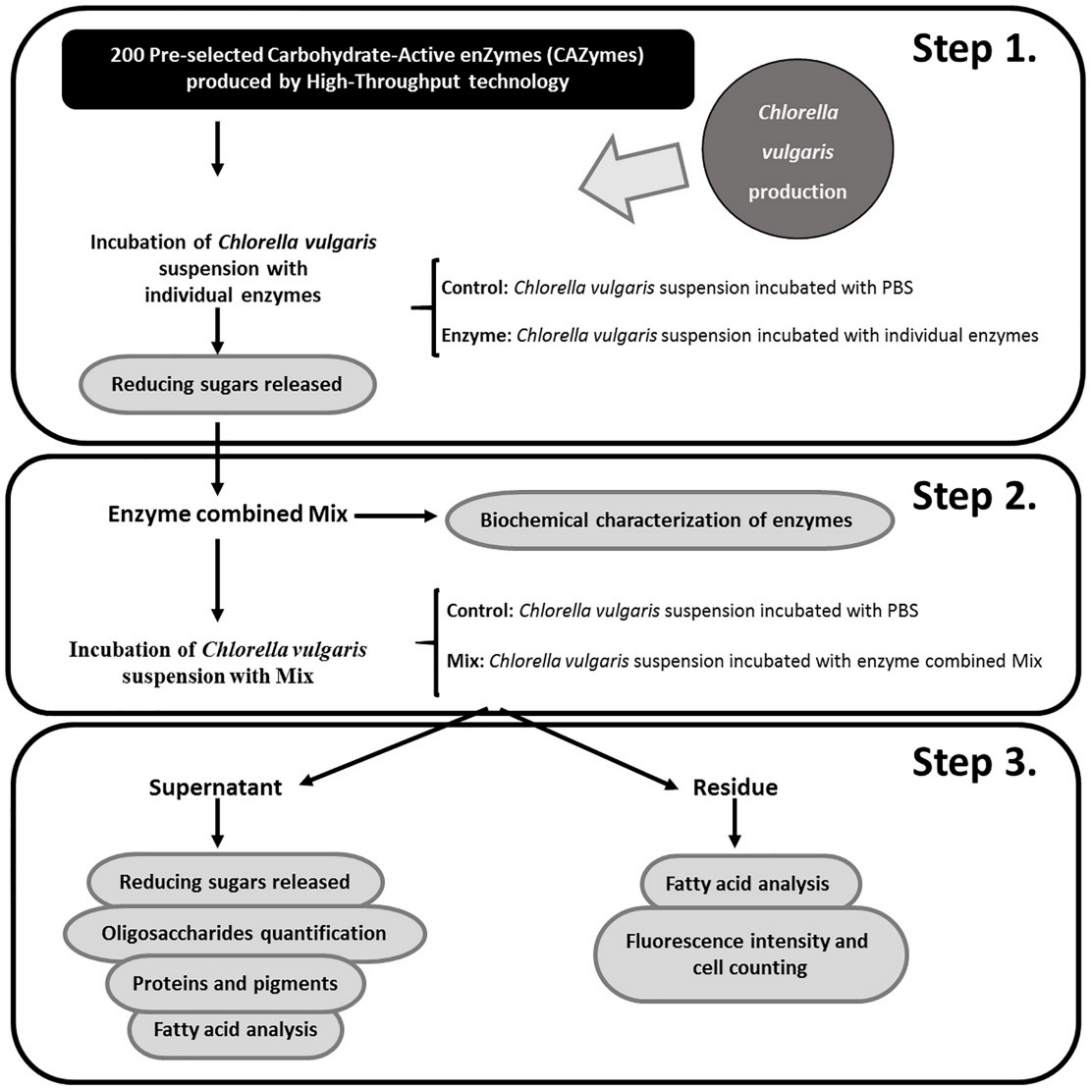
Illustration on the laboratory protocol followed by Coelho et al. [9, 10] to disclosure novel combination of feed enzymes to disrupt *Chlorella vulgaris* cell wall. Briefly, after *Chlorella vulgaris* cultivation, this flow chart depicts: as step 1, the individual screening of the most functional enzymes on disrupting *Chlorella vulgaris* cell wall; as step 2, finding the best CAZymes cocktail to disrupt *Chlorella vulgaris* cell wall; and as step 3, assessing the degree on *Chlorella vulgaris* cell wall disruption by measuring the amount of reducing sugars released, fatty acid analysis, oligosaccharides quantification, proteins, pigments with antioxidant function, and fluorescence intensity as well as cell counting.

**Fig 2:**
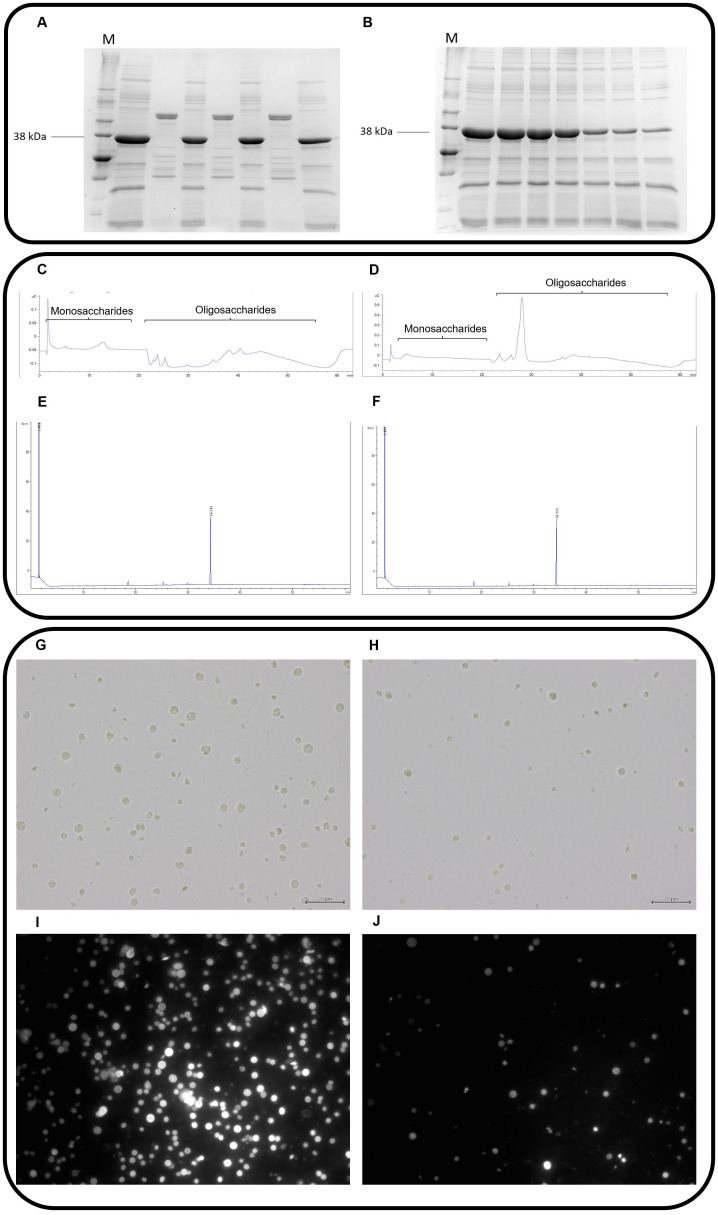
Illustrative images from raw results of each methodology applied across steps 2 and 3 of the laboratory protocol to disrupt *Chlorella vulgaris* cell wall. A) SDS-PAGE gels visualization to describe proteolysis experiments. M–molecular marker, 38 kilodaltons corresponds to peptidoglycan N-acetylmuramic acid deacetylase enzyme. Wells 1, 3 and 7 correspond to the control. Wells 2, 4 and 6 correspond to the incubation of the enzyme with pancreatin at time intervals of 15 min, 30 min and 1 hour, respectively; B) SDS-PAGE gels visualization to describe thermostability experiments. M–molecular marker, 38 kilodaltons corresponds to peptidoglycan N-acetylmuramic acid deacetylase enzyme. Wells 1–7 correspond to the incubation of the enzyme at 30 °C to 55 °C; C-D) HPLC chromatogram to exemplify mono- and oligosaccharides quantification in the control and in the mixture treatment, respectively; E-F) GC chromatogram to exemplify fatty acids quantification in the control and in the mixture treatment, respectively;; G-H) bright-field microscope observation for cell counting in the control and in the mixture treatment, respectively (×400; scale bar: 20 μm); I-J) fluorescence intensity for *Chlorella vulgaris* cell wall disruption in the control and in the mixture treatment, respectively (×400).

The first step on the design of a successful laboratory protocol to disclosure novel feed enzymes to break up *C*. *vulgaris* cell wall is to cultivate this species (Fig 1). At this stage, it is imperative to fully characterize *C*. *vulgaris* cell wall composition. Following our own hypothesis that nutrients availability is largely enhanced after disruption of microalga cell wall, a vast library containing CAZymes, including glycoside hydrolases, glycosyl transferases, polysaccharide lyases and carbohydrate esterases, and sulfatases, with well-defined and wisely thought-out enzymatic properties, are established by recombinant expression in *Escherichia coli* bacteria. The CAZy database (http://www.cazy.org/) presents complete and detailed information about the described CAZymes. These enzymes are naturally chosen, according to the composition of the matrix of insoluble carbohydrates of *C*. *vulgaris* cell wall, comprising glucosamine, galactose, rhamnose, mannose or alginates [5]. This is actually a pressure point on the lab strategy designed by Coelho et al. [9, 10]. The selected CAZymes should then be produced in a high-throughput (HTP) platform, including gene synthesis and cloning as well as protein expression and purification, allowing a faster and more efficient enzyme production when compared to the traditional method. The gene synthesis and protein production through a HTP platform are technologies that were developed and implemented by Nzytech (Lisbon, Portugal). However, the individual CAZymes can be commercially also obtained from biotechnology companies, such as Nzytech (Lisbon, Portugal), Sigma-Aldrich (St. Louis, Missouri, USA), Megazyme (Leinster, Ireland) and Prozomix Limited (Northumberland, England, UK) in order to be applied in any microalgae cell wall disruption protocol. Afterwards, the individual screening of the aforementioned enzymes to assess possible disruption of *C*. *vulgaris* cell wall is the goal. Still in step 1, pre-washed *C*. *vulgaris* is incubated, overnight, at 20 mg/mL and each individual enzyme at 20 μg/mL, versus a control, defined as the microalgae suspension incubated with PBS 1×, in triplicate, followed by the measurement of the amount of reducing sugars released using the classical 5-dinitrosalicylic acid (DNSA) protocol [12] (Fig 1). This protocol represents a quick and reliable method to perform a rapid screening of the activity rate in the *C*. *vulgaris* cell wall, of each one of the 200 CAZymes that compose the large enzyme library, aiming to classify the CAZymes according to its cell wall disruption ability. The microalgae concentration was fixed and defined according to a previous protocol of microalgae cell wall disruption [13]. The concentration of enzymes was defined in such a way that there is no immediate saturation of the enzymes. Moreover, the enzymatic reaction occurs throughout the incubation time, without wasting enzyme. During the incubations, enzymatic denaturation was not observed. Microalgae prewashing is a critical step to avoid the appearance of artefacts in the DNSA protocol. In step 2, a small selection of individual recombinant CAZymes is obtained from the step 1 as able to disrupt, to some extent, *C*. *vulgaris* cell wall (Fig 1). The criteria for this selection are: the rate of reducing sugars released in step 1; the main substrates of enzymes, linking to available information about *C*. *vulgaris* cell wall composition; and enzyme production yields. Thus, the selected enzymes displayed activity in the individual screening (step 1), with enzymatic activities according to *C*. *vulgaris* cell wall composition, and with good rates of recombinant production. Then, these enzymes are tested, in triplicate, in combination at 20 μg/mL in a ratio of 1:1:1:1 to obtain the maximum possible yield of cell wall disruption due to an enzymatic synergistic effect (in parallel with running the control). At this stage, it is also important to characterize biochemically each selected enzyme present in the mixture, by testing thermostability and resistance to proteolysis [9, 10]. One by one, each enzyme from the mixture is subjected to 12 different temperature conditions (without incubation and with incubation at 30 °C, 37 °C and 40 °C to 80 °C at 5 °C intervals) for 30 min. Then, the incubation is cooled on ice for 10 min and centrifuged at 16,100 *g* for 8 min at 4 °C. The supernatant is recovered and the protein amount is quantified using a NanoDrop 2000/2000c. To validate results, the supernatants are also analysed by 14% SDS-PAGE gels (Fig 2B). In order to check the proteolytic action of pancreatin to which feed enzymes are regularly exposed in the gastrointestinal tract of animals [14, 15], our target enzymes are treated with pancreatin at 37 °C. Each enzyme, at a concentration of 1 g/L, is subjected to the proteolytic action of pancreatin, which is incubated at a final concentration of 2.5 g/L. The reactions are incubated at 37 °C, at regular intervals of 15 min for 120 min. The results are presented at periods of 15, 30, 60, 90 and 120 min of incubation for each enzyme. The qualitative scale on proteolysis resistance is based on SDS-PAGE gels visualization: −, no resistant (only fragmentation bands); +, partially resistant (protein and fragmentation bands) (Fig 2A).

As a result of combining enzymes, an enzyme mixture or cocktail composed by exo-β-glucosaminidase, alginate lyase, peptidoglycan N-acetylmuramic acid deacetylase and lysozyme demonstrated to have potential to disrupt *C*. *vulgaris* cell wall. This enzyme mixture is applied from now on. In step 3, the degree of microalga cell wall disruption induced by the enzymatic cocktail is assessed in the residue by optical and fluorescence microscopy, for cell counting using a Neubauer chamber by direct observation on a bright-field microscope for both control and CAZymes cocktail treatments (Fig 2G and 2H, respectively), and for cell wall rupture by fluorescence intensity, after staining with Calcofluor White for both control and CAZymes cocktail treatments, on a fluorescence microscopy, respectively (Fig 2I and 2J, respectively). The fluorescence intensity is quantified using the ImageJ software and it is measured in 13 slides for the control and in 13 slides for the mixture treatment to ensure an adequate number of replicates to apply the statistical test. These measurements are complemented with the quantification of fatty acids using a gas chromatograph coupled with flame ionization detector (GC-FID) applying fatty acids extraction procedures of Folch et al. [16] and Carlson [17], after incubation with the enzymatic mixture treatment. Then, fatty acids are esterified to methyl esters (FAME) by acid catalysis with acetylchloride-methanol solution at 80 °C for 60 min, as described by Batista et al. [18]. In the supernatant, the amount of reducing sugars released and the oligosaccharides profile are quantified, by the DNSA method [12] and high performance liquid chromatography (HPLC), equipped with an electrochemical detector (ECD), correspondingly. Briefly, the resolution of mono and oligosaccharides is achieved using a Dionex CarboPac PA10 column (4 × 250 mm, Thermo Fisher Scientific Inc, USA) fitted to a CarboPac PA10 guard column (4 × 50 mm) and a mobile phase with a flow rate of 1 mL/min for 60 min at 25 °C, as follows: isocratic elution with 18 mM NaOH (eluent A) during 18 min, gradient with 100–0 mM NaOH (eluent B) and 0–75 mM sodium acetate in 100 mM NaOH (eluent C) from 18–40 min, and re-equilibration to 18 mM NaOH during 20 min. This HPLC method was based on the procedure described by Thermo Fisher Scientific [19] and optimized by our research group [9, 10]. In order to identify the retention time of mono and oligosaccharides, a standard chromatogram was performed using the following sugar standards: glucose, cellobiose, cellotriose, cellotetrose, cellopentose and cellohexose. The quantification of total oligosaccharides is based on a standard curve, using a range of concentrations from 0.025 mM to 0.2 mM of glucose (Fig 2C and 2D). The results are expressed as equivalent moles of glucose released per gram of microalga. The extraction of inner bioactive compounds with nutritional interest is concluded with the quantification of proteins by using the Kjeldahl method [20], diverse pigments with antioxidant function (such as chlorophylls a and b, and carotenoids) by following Hynstova et al. [21] protocol as well as the fatty acid profile using GC-FID according to the methodology described above, after incubation with the enzymatic mixture treatment (Fig 2E and 2F). In each round of experiments, the control is defined as the microalgae suspension incubated with phosphate buffered saline (PBS) 1× (a non-toxic buffer solution commonly used in biological research. Unlike water, PBS prevents cells rupturing or shrivelling up due to osmosis).

### 2.2. Other enzymatic strategies to break up microalgae cell wall

The enzymatic lysis displays numerous advantages as the selected method for the disruption of microalgae cell wall. That is why highly valued research teams have been working worldwide on this topic looking for the most possible economical solution [22, 23]. Zheng and colleagues [24] tested the effectiveness of different lysis methods using three enzymes individually, a snailase, a lysozyme and a cellulase on *C*. *vulgaris* microalga. To measure the degree of cell wall degradation after each enzyme action, the lipid extraction yield was quantified, and the three enzymes were found as effective. In a similar study, Cho et al. [25] evaluated the power of cellulases and β-glucosidases combined towards the disruption of *C*. *vulgaris* cell wall. These authors assessed the disruption degree of cell wall through the lipid extraction yield. In a study conducted by Gerken et al. [26], the enzymatic cell wall degradation of different microalgae strains, *Chlorella* and *Nannochloropsis*, were tested. To exploit the enzymatic activity against microalgae cell wall, the authors applied a growth inhibition assay, in which microalgae were cultivated in the presence of different enzymes, individually or combined. The inhibition of microalgae growth suggests that the enzyme is degrading the cell wall during construction. Then, the authors measured the permeability percentage of microalgae derived from the enzymatic action in the cell wall through a flow cytometer coupled with imaging. The enzymatic action increases the permeability of microalga leaving DNA into the extracellular space, which is detected by flow cytometry. Finally, through electron microscopy, it was possible to identify the extent of cell wall damage promoted by the enzymes [26].

Although the enzymatic methodologies of cell wall disruption are very promising, they display many disadvantages. One of them is the prohibitive cost. This is directly linked to the fact that enzymes cannot be generally recovered after being used [22]. A possible resolution on this problem was originally introduced by Fu et al. [13] through the immobilization technology applied to cellulase onto an electrospun polyacrylonitrile (PAN) nanofibrous membrane. In this sense, in addition to achieve appreciable rates of microalgae cell wall degradation and an improvement on microalgae lipid extraction yield, it was possible to re-use enzymes and to reduce the amount needed.

More recently, several studies on the development of enzymatic strategies to promote the disruption of *C*. *vulgaris* cell wall were published in the literature. In order to overcome the expensive cost of commercial enzymes, Dwi et al. [27], studied the capacity of extracellular cellulase and hemicellulase produced by *Bacillus licheniformis* from Milkfish Gut. The authors analyzed the optimal conditions of enzyme production by *Bacillus licheniformis* and then, through a simple incubation *C*. *vulgaris*/enzymes evaluated the degradation of the wall under a binocular inverted microscope and concluded that these enzymes were capable of degrading *C*. *vulgaris* cell walls [27]. Finally, Canelli et al. [28] developed a similar strategy to ours to identify an enzyme mixture capable of degrading *C*. *vulgaris* cell wall. The authors performed an individual screening of enzymes based on disruption efficacy (total carbon and total nitrogen release) during the incubation. Chitinase, rhamnohydrolase, and galactanase displayed the best results on the individual screening. The next step was to perform an incubation with *C*. *vulgaris* and a mixture of these enzymes, evaluating its ability to degrade the cell wall through the bioaccessibility of lipid and protein, measurement of the particle size of cells, and observation on a light microscope [28].

## 3. Future outlook and challenges

Microalgae can provide a sustainable and environmentally-friendly solution to fight food and energy crisis that our planet is currently facing. The best possible knowledge on the physicochemical characterization of microalgae is paramount, as it allows selecting which microalgae species are the best for different biotechnological applications and scientific purposes [21]. Top research and development initiative are presently directed to increase the yield extraction of microalgae inner nutritional products and, at the same time, to reduce the overall operational cost [27–29]. The complexity of microalgae cell wall is largely determined by microalga species, growth condition and phase, as well as the existence of stress factors. Short- and medium-term future research should be focused on a better understanding upon the binomial interaction microalga/enzyme cocktail and on the specific mechanisms of algae cell lysis, both important breakthroughs to reduce the cost of microalgae cell wall disruption [28, 30], and on the development of new strategies that turn cell wall degradation into a more effective and economical process. An all-inclusive multidisciplinary approach having chemists, biotechnological engineers and biologists as partners with increased know-how on improved selectivity, better shelf-life, efficiency, specificity, and economic viability, would benefit this goal.

## Supporting information

S1 File Step-by-step protocol on a successful lab protocol to disrupt *Chlorella vulgaris* microalga cell wall. (PDF)
