# Supplementary material for: Testimony on a successful lab protocol to disrupt Chlorella vulgaris microalga cell wall
Source: PLoS One. 2022 May 19;17(5):e0268565. doi: 10.1371/journal.pone.0268565 (PMC9119475; doi:10.1371/journal.pone.0268565)
Supplement: S1 File — (PDF) [file pone.0268565.s001.pdf]

Testimony on a successful lab protocol to disrupt Chlorella vulgaris microalga cell wall

Diogo Coelho[1], Paula A. Lopes[1], José A. M. Prates[1]

[1]CIISA - Centro de Investigação Interdisciplinar em Sanidade Animal and Laboratório Associado para Ciência Animal e Veterinária (AL4AnimalS), Faculdade de Medicina Veterinária, Universidade de Lisboa, Avenida da Universidade Técnica, Pólo Universitário do Alto da Ajuda, 1300-477 Lisboa, Portugal

dx.doi.org/10.17504/protocols.io.dm6gpb9z5lzp/v1

Diogo Coelho

Over the last decades, microalgae have gained popularity due to demand for novel environmental green solutions and development of innovative mass-production sources for multiple processes, including animal feed and human diet, turningmicroalgae into an exquisite candidate for several ecofriendly technologies. Notwithstanding, there is a catch. Most species of microalgae, as the case of common *Chlorella vulgaris* (*C. vulgaris*)display a recalcitrant cell wall, characterized by a complex matrix of polysaccharides and glycoproteins, which constitutes a major barrier for monogastric species digestibility and extraction of inner valuable nutritional compounds. To overcome this limitation, the development of feed enzymes, in particular Carbohydrate-Active enZymes (CAZymes) with capacity to disrupt *C. vulgaris*cell wallmay contribute to improve the bioavailability of these microalgae compounds in monogastric diets, namely at high levels of incorporation. In order to disclosure novel combination of feed enzymes to disrupt *C. vulgaris* cell wall, a lab protocol was implemented by our research team containingthe following key steps: after microalgae cultivation and having available a repertoire of two hundred pre-selected CAZymes produced by high-throughput technology, thestep 1 is the individual screening of the most functional enzymes on disrupting *C. vulgaris*cell wall (versus a control, defined as the microalgae suspension incubated with PBS) and the determination of reducing sugars released by the 3,5-dinitrosalicylic acid (DNSA) method; step 2 concerns on finding the best CAZymes cocktail, testing the synergistic effect of enzymes, to disrupt *C. vulgaris*cell wall (in parallel with running the control) along with characterization of each enzyme thermostability and resistance to proteolytic attack, to which feed enzymes are subjected in the animal gastrointestinal tract; step 3 is the assessment of *C. vulgaris* cell wall degradation degree by measuring the amount of reducing sugars released by the DNSA method, fatty acid analysis by gas chromatography (GC) with flame ionization detector(FID), oligosaccharides quantification byhigh performance liquid chromatography(HPLC) equipped with an electrochemical detector (ECD),protein content by the Kjeldahl method, and various pigments (chlorophylls a and b, and total carotenoids) in the supernatant. In the correspondent residue, we also assessed cellular counting using a Neubauer chamber by direct observation on a bright-field microscope and fluorescence intensity, after staining with Calcofluor White for both control and CAZymes cocktail treatments, on a fluorescence microscope. Beyond animal feed industry with impact on human nutrition, our lab protocol may increase the yield in obtaining valued constituents from *C. vulgaris*microalga for other biotechnological industries.

DOI

dx.doi.org/10.17504/protocols.io.dm6gpb9z5lzp/v1

Diogo Coelho, Paula A. Lopes, José A. M. Prates . Testimony on a successful lab protocol to disrupt Chlorella vulgaris microalga cell wall. **protocols.io** https://dx.doi.org/10.17504/protocols.io.dm6gpb9z5lzp/v1

protocol ,

Mar 30, 2022

Mar 30, 2022

60085

Construction of the CAZymes bank and production of microalgae Incubation of C. vulgaris suspension with individual CAZymes (glycoside hydrolases, glycosyl transferases, polysaccharide lyases and carbohydrate esterases)

1 Preparation of *C. vulgaris* suspension (20 mg/mL) resuspending the microalgae powder in PBS buffer and mix with vortex.

2 Prewash the *C. vulgaris* suspension by incubating it in an orbital shaker at 37ºC, 150 rpm for 30 minutes. Centrifuge the suspension at 4000 *g* for 30 minutes. Discard the supernatant and resuspend the pellet in new PBS buffer (20 mg/mL) and mix with vortex.

3 In a 24-well plate, add 1 mL of *C. vulgaris* suspension to each well. Then, add the respective enzyme to each well to a final concentration of 20 µg/mL. In the corresponding control wells, add 50 µL of PBS buffer instead of the enzyme. The incubations should be performed in triplicate.

4 Seal the plate and incubate in an orbital shaker at 37ºC, 60 rpm, overnight.

5 Then, centrifuge the plate at 4000 *g* for 15 minutes and recovery the supernatant to an eppendorf.

6 Boil the samples for 5 minutes and centrifuge at 10000 *g* for 5 minutes.

7 Recovery the supernatant for a new eppendorf and proceed to the determination of reducing sugars release.

Reducing sugars release determination (DNSA method)

8 Add to a new eppendorf 600 µL of the supernatant from the stage 7. Add to the supernatant 600 µL of DNSA reagent previously prepared (**Note 1**).

8.1 **Note 1:** DNSA reagent – 1% 3,5-Dinitrosalicylic acid; 0.2% phenol and 1% sodium hydroxide. Before use, add 200 µL of sodium sulphite (5%) and 2 µL of glucose (20%) per 20 mL of DNSA reagen (Miller, 1959).

9 Mix vigorously and incubate in boiling water at 100ºC for 15 minutes.

10 Place the eppendorfs on ice for 5 minutes.

11 Measure the absorbance of samples (λ=570 nm).

12 The amount of reducing sugars released is calculated using a calibration curve with sequential dilutions of a glucose solution with an initial concentration of 1 mg/mL and absorbance determined through the DNSA method (Miller, 1959).

Incubation of C. vulgaris suspension with the enzyme mix selected composed by exo-β-glucosaminidase, alginate lyase, peptidoglycan N-acetylmuramic acid deacetylase and lysozyme

13 Repeat stages 1 to 7. At stage 3, add the enzyme mix instead of individual enzymes. The final concentration of each enzyme in the mix is 20 µg/mL in a ratio of 1:1:1:1.

Biochemical characterization of enzymes

14 Thermostability analysis – Incubate each enzyme of the mix in a defined final concentration at the following temperatures: 30 °C, 37 °C and 40 °C to 80 °C at 5 °C intervals for 30 minutes and perform a control (no incubation). Then, cool the incubation on ice for 10 min and centrifuged at 16,100 *g* for 8 min at 4 °C. Recover the supernatant and quantify the protein amount in triplicate using a NanoDrop. To validate the results, analyse the supernatants by performing 14% SDS-PAGE gels.

15 Proteolysis resistance – Place 200 µL of each enzyme that compose the mix, at a concentration of 1 g/L in individual eppendorfs. For each enzyme, there is a control and a treatment: 200 µL of PBS are added for the control; 200 µL of porcine pancreatin at 5 g/L are added for the treated sample. Incubate the reactions at 37 °C during regular 15 min intervals until 2 hours. Remove the samples and perform 14% SDS-PAGE gels to analyse results.

Analysis on the supernatant - Reducing sugars release determination (DNSA method)

16 Repeat stages 8 to 12.

Analysis on the supernatant - Fatty acids analysis

17 1 mL of the supernatants is lyophilized for 24 hours.

18 It should be applied the method of Folch et al. (1957) by replacing chloroform:methanol (2:1, v/v) by dichloromethane:methanol (2:1, v/v), according to Carlson (1985) to perform the lipid extraction from supernatants.

19 Perform the esterification of fatty acids to fatty acids methyl esters (FAME) using acid catalysis with acetylchloride-methanol solution at 80 °C for 60 min, as described by Batista et al. (2013).

20 Proceed to the analysis of FAME as described by Coelho et al. (2019).

Analysis on the supernatant - Oligosaccharides quantification

21 The quantification of oligosaccharides in the supernatants is performed by HPLC. 10 µL of supernatants are injected directly into the HPLC equipment.

22 The analysis of the oligosaccharides profile is made using a Dionex CarboPac PA10 column and electrochemical detector (ECD), according to the manufacturer's instruction method (Thermo Scientific. Dionex CarboPac PA10 in Column Product Manual).

23 The quantification of total oligosaccharides in the supernatant is based on a standard curve, using a range of concentrations from 0.025 mM to 0.2 mM of glucose.

Analysis on the supernatant - Protein quantification

24 The determination of protein content is carried out by applying the classic Kjeldahl method (AOAC, 2000).

Analysis on the supernatant - Pigments quantification

25 The determination of pigments with antioxidant function (as chlorophylls a and b, andcarotenoids) is achieved by following Hynstova et al.(2018) protocol.

Analysis on the residue

26 After the plate centrifugation (stage 5), the microalgae residue is recovered.

Analysis on the residue - Fatty acids analysis

27 Lyophilize 10 mg of residues for 24 hours.

28 Repeat stages 18 to 20.

Analysis on the residue - Cell counting

29 Resuspend 10 mg of residue in 500 µL of PBS buffer. Add another 2 mL of PBS buffer and homogenise properly by performing “up and down” movements with the micropipette.

30 Add 20 µL of the homogenate to a Neubauer chamber.

31 Place the chamber in an optical microscope and perform cell counting.

Analysis on the residue - Fluorescence intensity quantification

32 Add 10 µL of the homogenate prepared at stage 25 to a microscope slide.

33 Then, add 2 µL of Calcofluor White stain and 2 µL of potassium hydroxide (10%) to the sample.

34 Cover with the coverslip and wait 1 minute.

35 Observe in the fluorescence microscope at magnification of 400× using the DAPI filter.

36 Perform the quantification of fluorescence intensity with the Image J software.
